# Melatonin Induces Parthenocarpy by Regulating Genes in Gibberellin Pathways of ‘Starkrimson’ Pear (*Pyrus communis* L.)

**DOI:** 10.3389/fpls.2018.00946

**Published:** 2018-07-04

**Authors:** Jianlong Liu, Rui Zhai, Fengxia Liu, Yingxiao Zhao, Huibin Wang, Lulu Liu, Chengquan Yang, Zhigang Wang, Fengwang Ma, Lingfei Xu

**Affiliations:** College of Horticulture, Northwest A&F University, Yangling, China

**Keywords:** melatonin, pear, parthenocarpy, gibberellin, cell division, cell expansion, transcriptome analysis

## Abstract

Parthenocarpy, the production of seedless fruit without fertilization, has a variety of valuable qualities, especially for self-incompatible species, such as pear. To explore whether melatonin (MT) induces parthenocarpy, we used ‘Starkrimson’ pear as a material for morphological observations. According to our results, exogenous MT promoted the expansion and division of the mesocarp cells in a manner similar to hand pollination. However, the seeds of exogenous MT-treated fruit were undeveloped and aborted later in the fruit-setting stage. To further investigate how MT induced parthenocarpy, we studied changes of related hormones in the ovaries and found that MT significantly increased the contents of the gibberellins (GAs) GA_3_ and GA_4_. Thus, paclobutrazol (PAC), a GA-biosynthesis inhibitor, was used to study the relationship between GAs and MT. In addition, spraying MT after treatment with PAC did not increase GA content nor lead to parthenocarpy. Through a transcriptome analysis, we discovered that MT can cause significant upregulation of *PbGA20ox* and downregulation of *PbGA2ox*. However, no significant difference was observed in *PbGA2ox* compared with the control after PAC and MT applications. Thus, MT induces parthenocarpy by promoting GA biosynthesis along with cell division and mesocarp expansion in pear.

## Introduction

Parthenocarpy, the production of seedless fruit, induces fruit development naturally or artificially without the fertilization of ovules (Gustafson, 1942). As an important cultivated fruit worldwide, most pears are self-unfruitful because of their gametophytic self-incompatibility. Without pollination, the trees can only generate fruit by parthenocarpy (Spena and Rotino, 2001). Determining an efficient method to produce pear fruit by parthenocarpy is therefore important.

Plant hormones affect fruit set, growth and development (Srivastava and Handa, 2005). Parthenocarpy can be induced by exogenous applications of plant hormones. Most genes involved in fruit set are related to growth regulators of fruit development, such as gibberellins (GAs) and auxins (Ozga and Reinecke, 2003). Auxin indole-3-acetic acid (IAA) can also induce parthenocarpy in many horticultural plants, such as tomato, cucumber, and zucchini (Martinelli et al., 2009; Pomares-Viciana et al., 2017). In addition, parthenocarpy can be controlled by a single gene, such as a transcriptional factor or a receptor in phytohormone signaling pathways (Martí et al., 2007; Serrani et al., 2010; Fuentes et al., 2012). Although two important auxin-responsive gene families, auxin response factor (ARF) and AUX/IAA, are related to the development of parthenocarpic fruit in *Arabidopsis thaliana* and *Solanum lycopersicum* (tomato) (Kumar et al., 2011). Dorcey et al. (2009) found that GA is downstream of auxin in the regulatory process of parthenocarpy in these two species. GAs can induce parthenocarpy in many fruit trees, such as apple (Watanabe et al., 2008), loquat (Aslmoshtaghi and Shahsavar, 2013), peach (Crane et al., 1960), and pear (Niu et al., 2015). The GA content of the parthenocarpic citrus variety ‘Satsuma’ is higher than that of the non-parthenocarpic ‘Clementine,’ which indicates that endogenous GA promotes parthenocarpic development (Talon et al., 1992). Overexpression of the GA 20-oxidase (GA2ox) gene *CcGA20ox1* from the citrange ‘Carrizo’ (*Citrus sinensis* L. Osbeck ×*Poncirus trifoliata* L. Raf.) aids the development of parthenocarpic fruits in tomato (Greco et al., 2012). GA2oxs are catabolic enzymes that deactivate active Gas. In one study, the silencing of five *GA2ox* genes in transgenic tomato plants resulted in a significant increase in their GA_4_ content and ability to undergo parthenocarpy (Martínez-bello et al., 2015).

Melatonin (MT) is an important plant growth regulator that can improve resistance to biotic and abiotic stresses, such as pathogen attack (Yin et al., 2013), extreme temperature (Tiryaki and Keles, 2012), excess copper (Posmyk et al., 2008), intense light (Tiryaki and Keles, 2012), salinity (Li et al., 2012), drought (Liu et al., 2015), and senescence (Wang et al., 2013). MT, which plays a major role in regulating plant rhythm and plant growth, is involved in root morphology, senescence, seed germination, crop yield, and fruit ripening (Arnao and Hernández-Ruiz, 2015; Reiter et al., 2015; Tan et al., 2015). These functions are similar to those of IAA in plants, and they have a common precursor, tryptophan. In addition, a low concentration of MT (10 μmol L^−1^) in growing plants can promote carbohydrate metabolism, photosynthesis, and sucrose loading and transportation in phloem, thus promoting plant growth; in contrast, a high MT concentration (1 mmol L^−1^) inhibits sucrose loading in phloem and promotes the accumulation of excess sugar, hexose, and starch in leaves. A feedback mechanism involving MT thus controls leaf photosynthesis and plant growth (Zhao et al., 2015). A concentration effect of MT on plant growth and photosynthesis has also been confirmed in cherry (Sarropoulou et al., 2012). In *Datura metel* ‘Mandala,’ MT content is highest in developing flower buds; it decreases during flower bud maturation but then increases during early fruit development (Murch et al., 2009). A similar result has been observed in tomato (Okazaki and Ezura, 2009). MT therefore likely has a specific role in plant reproduction and helps trigger a sexual to asexual transformation in plants. MT may thus induce parthenocarpy. Noteworthily, GA causes parthenocarpy in pear (Zhang et al., 2017). In addition, MT can regulate GA synthesis (Zhang et al., 2017) and stabilizes the GA downstream inhibitor DELLA (Shi et al., 2016). We therefore further speculate that MT causes parthenocarpy by regulating GA pathways.

To test the above hypothesis, we carried out histomorphological observations, high-performance liquid chromatography–tandem mass spectrometry (HPLC–MS/MS) and transcriptomics analyses of pear ovaries after MT treatments. Our results confirm that MT can induce parthenocarpy in ‘Starkrimson’ pear (*Pyrus communis* L.) and provide evidence that MT causes parthenocarpy by regulating GA pathways.

## Materials and Methods

### Plant Material, Growth Conditions, and Treatments

Experiments were carried out in a pear orchard located in Wugong, Shaanxi Province, China (34.12°N, 108.26°E). Wugong has a continental monsoon climate, with an average annual precipitation of 633.7 mm and an average annual temperature of 12.7°C. During anthesis, the average temperature was 10°C, with an average humidity of 71% and total rainfall of 45.68 mm. Four-year-old ‘Starkrimson’ pear (*P. communis* L.) grafted onto *Cydonia oblonga* rootstock were used as the experimental material. Two days before anthesis, all treated and control plants were bagged to prohibit pollination. Healthy and uniform plants were subjected to one of four treatments: (i) water on unpollinated flowers, serving as the non-pollination treatment (CK); (ii) a solution of 1 mmol L^−1^ IAA (IAA) [the concentration previously determined by de Jong et al. (2009)] sprayed on unpollinated ‘Starkrimson’ flowers at anthesis; (iii) a solution of 100 μmol L^−1^ MT (MT) (the concentration giving the highest fruit set rate in preliminary tests; Supplementary Figure S1), also sprayed on unpollinated ‘Starkrimson’ flowers at anthesis; and (iv) hand pollination, performed at the same time. Other healthy and uniform plants were assigned to two test conditions: (i) standard water supply or (ii) a water solution supplemented with 100 μmol L^−1^ PAC. For the two groups, solutions of 100 μmol L^−1^ MT were sprayed on unpollinated ‘Starkrimson’ flowers at anthesis, referred to as MT and MP, respectively (Supplementary Figure S2). A part of each sample was immediately fixed in formalin-aceto-alcohol for histological observation. After being rapidly frozen in liquid nitrogen, the other tissues were stored at −80°C.

### Determination of Fruit Set Rate

A total of 50 blooms on each pear tree were labeled and bagged immediately after treatment. At 20 days after anthesis (DAA), the bags were removed. The formula used to calculate the fruit set rate was as follows:

Fruit set rate (%)=(number of fruitlets remaining/30)×100%.

### Vascular Bundles Staining and Paraffin Sectioning Methods

For histological observations, stalks from fruit samples that were collected 10 DAA were immediately stained in alkaline magenta solution and observed under a stereoscopic microscope at specific timepoints.

Non-pollinated, hand pollinated, and MT-treated fruit samples were collected at 5 DAA, immediately fixed in formalin-aceto-alcohol solution and stored at 4°C (Phillips and Hayman, 1970). The ovaries were dehydrated in an ethanol and xylene series, embedded in paraffin, sectioned into 8-μm slices, dried, and stained with safranine and fast green (Lin et al., 2007). Anatomical images were observed using a microscopic imaging system (BX51+PD72+IX71, OLYMPUS, Japan). Cell areas were ascertained from mesocarp longitudinal sections (30 cells per section from 10 to 15 sections) using ImageJ software.

### Soluble Solid and Organic Acid Contents, Peduncle Length and Fruit Shape Index Analysis

The content of soluble solids was determined at 25°C using a portable system (Pocket Refractometer PAL-1, Atago, Japan). Organic acids were measured using a portable system (GMK-835F, G-WON, Korea) over a range of 0.0–3.5% with an accuracy of ± 0.05%. All measurements were performed on 10 replicates, each consisting of a single fruit.

A vernier caliper was used to measure peduncle length. The formula used to calculate fruit shape index was as follows:

Fruit shape index=fruit suture diameter/polar diameter.

### Phytohormone Analysis

Levels of GA_3_, GA_4_, IAA, and abscisic acid (ABA) were determined by HPLC–MS/MS. Approximately 0.5 g of ovaries was ground in a pre-cooled mortar containing 5 mL of extraction buffer composed of isopropanol and hydrochloric acid. The extract was shaken at 4°C for 30 min. Then, 10 mL of dichloromethane was added, and the sample was again shaken at 4°C for 30 min. The sample was then centrifuged at 18,000 ×*g* for 5 min at the same temperature, and the lower, organic phase was extracted. The organic phase was dried under N_2_, dissolved in 150 μL methanol (0.1% methane acid) and filtered through a 0.22-μm filter membrane. The purified product was then subjected to HPLC–MS/MS analysis. The HPLC analysis was performed using a ZORBAX SB-C18 (Agilent Technologies, United States) column (2.1 mm × 150 mm; 3.5 mm). The mobile phase A solvent consisted of methanol and 0.1% methanoic acid, and the mobile phase B solvent consisted of ultrapure water and 0.1% methanoic acid. The injection volume was 2 μL. MS conditions were as follows: a spray voltage of 4,500 V, and air curtain, nebulizer, and auxin gas pressures of 15, 65, and 70 psi, respectively. The atomizing temperature was 400°C. Each sample consisted of three replicates from independent experiments.

### Transcriptome Analysis

Ovaries for RNA sequencing were collected from unpollinated, hand pollinated, MT-treated (without pollination), and MP-treated ‘Starkrimson’ at 5 DAA. Three independent biological replications were sequenced and analyzed.

A total of 3 μg RNA per sample was used as input material for RNA sample preparations. Sequencing libraries were generated using a NEBNext^®^ Ultra^TM^ RNA Library Prep Kit for Illumina^®^ (NEB, United States) following manufacturer’s recommendations, and index codes were added to attribute sequences to each sample. The clustering of the index-coded samples was performed on a cBot Cluster Generation System using a TruSeq PE Cluster Kit v3-cBot-HS (Illumina). After cluster generation, the library preparations were sequenced on an Illumina HiSeq platform and 125-bp/150-bp paired-end reads were generated. Raw data (raw reads) in FASTQ format were first processed using an in-house Perl script. In this step, clean data (clean reads) were obtained by removing reads containing adapters, reads containing poly-N and low-quality reads from the raw data. All downstream analyses were based on clean data of high quality. The index of the reference genome was built using Bowtie v2.2.3, and paired-end clean reads were aligned to the reference genome using TopHat v2.0.12. HTSeq v0.6.1 was used to count the read numbers mapped to each gene.

The resulting *P*-values were adjusted using Benjamini and Hochberg’s approach for controlling the false discovery rate. Genes with an adjusted *P*-value < 0.05 according to DESeq were considered to be differentially expressed. Genes were annotated using the ‘Dangshansuli’ database^1^ as a reference.

### Gene Expression Assessed by Quantitative Real-Time PCR (qRT-PCR)

Quantitative real-time PCR was performed on an ABI instrument using a SYBR Premix Ex Taq kit (Takara). The cDNA template was reverse transcribed using total RNA extracted from the five treatments at 5 DAA. Actin was used as an internal reference for the gene expression analysis. Primers for selected genes were designed using Primer Premier 5.0 software. The PCR amplification were carried out using the following program: initial incubation at 95°C for 30 s, followed by 40 cycles at 95°C for 5 s and 60°C for 30 s. Primers are listed in Supplementary Table S1. All reactions had triple biological repeats.

### Statistical Methods

Data were statistically analyzed by analysis of variance and tested for significant (*P* < 0.05) treatment differences using Duncan’s test. Results are presented as means ± standard deviation (SD) of three replicate samples.

## Results

**FIGURE 1:**
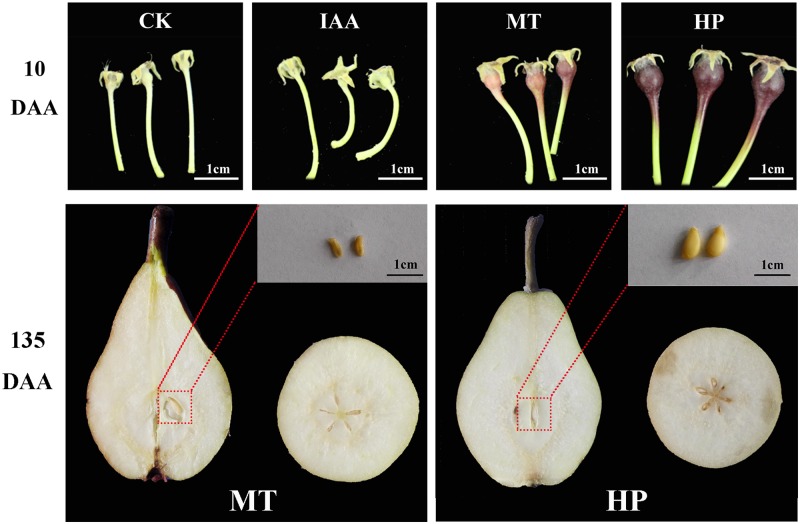
Effects of melatonin on fruit shape and seed development compared with other treatments. CK, control; IAA, indole-3-acetic acid treatment; MT, melatonin treatment; HP, hand pollination.

To investigate the effects of MT on fruit set, pear plant at the full-bloom stage were subjected to four treatments. Non-pollination-treated and IAA-treated ovaries did not develop normally, but the MT-treated ovaries were similar in size to those that were hand pollinated at 10 DAA (**Figure 1**). Hand pollination led to normal seeded fruits, while, MT-treated ovaries developed normal fruits without seeds (**Figure 1**). Fruits underwent apoptosis on the 10th day, and vascular tissues disintegrated in unpollinated carpopodia, resulting in an inability to transport nutrients normally. Vascular bundles developed normally in MT and hand-pollinated carpopodia, and the ovaries could be stained successfully with basic fuchsin staining for 30 min (Supplementary Figure S3). During the post-harvest storage period, MT had no significant effects on organic acid and soluble solid contents of fruits but significantly increased peduncle length and fruit shape index. For the fruit shape index, MT increased the fruit’s longitudinal diameter and shortened the fruit’s equatorial diameter, resulting in more slender fruit (Supplementary Figure S4). This result suggests that MT did not alter fruit flavor, like parthenocarpy in pear, but did change fruit appearance.

**FIGURE 2:**
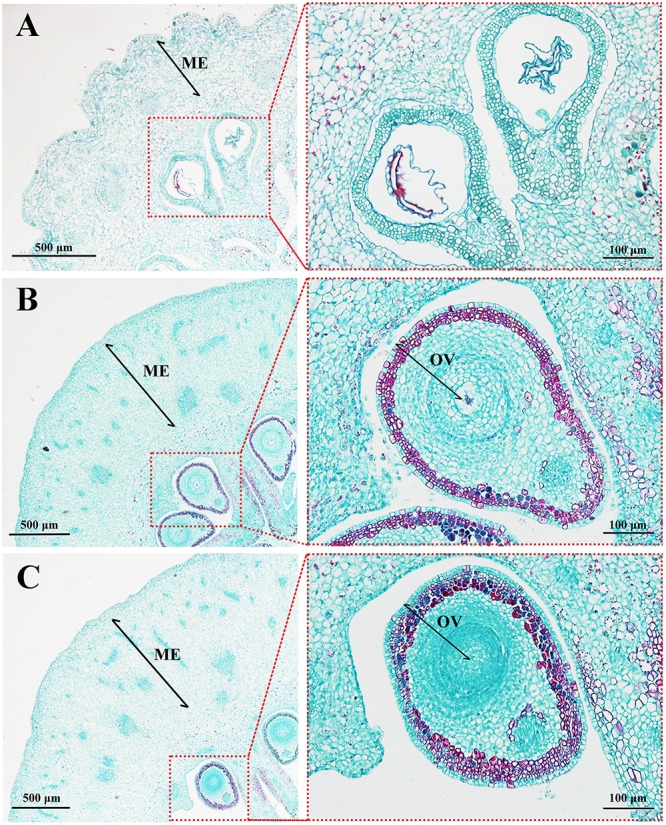
Histological observations of ‘Starkrimson’ pericarps 5 days after anthesis (DAA). **(A)** CK, control; **(B)** MT, melatonin treatment; and **(C)** HP, hand pollination. ME, mesocarp; OV, ovule.

To observe how MT causes fruit elongation, histological observations of fruit tissues were performed at early developmental stages. Mesocarp, the edible part of the fruit, comprised up to 31 cell layers after MT treatment, while that of the CK group consisted of ∼17 cell layers. Likewise, the area of mesocarp cells in MT ovaries was larger than in the CK, but there was no significant difference between the sizes of the MT and HP ovaries (**Figure 2** and **Table 1**). In the absence of pollination and fertilization, however, MT did not increase the cell division of seeds compared with that in the CK. The number of ovular cell layers was much lower than the 22 layers found after hand pollination (**Figure 2** and **Table 1**). MT thus can promote cell division and expansion, but not normal ovular development.

**Table 1:** Number of mesocarp and ovule layers, and corresponding cell sizes.

Treatments	Cellular layers		Cell area (μm^2^)	
	Mesocarp	Ovule	Mesocarp	Ovule
CK	17.75 ± 2.63b	–	553.95 ± 43.28b	–
MT	31 ± 0.81a	15.25 ± 0.50b	823.58 ± 83.73a	108.46 ± 28.30a
HP	33 ± 3.36a	22.50 ± 1.91a	772.46 ± 71.32a	95.25 ± 21.50a

*Cell area values are means of three replicates, with 30 cells per replicate (± SD). Different letters within a column indicate significant differences at P < 0.05 (Duncan’s range test).*

**FIGURE 3:**
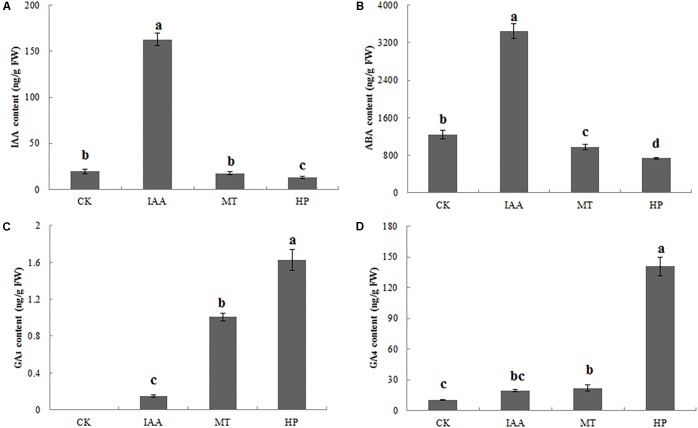
Indole-3-acetic acid (IAA), abscisic acid (ABA), and gibberellin (GA_3_ and GA_4_) contents of pear ovaries 5 DAA following different treatments. **(A)** IAA content, **(B)** ABA content, **(C)** GA_3_ content, and **(D)** GA_4_ content. The results are means ± SD (*n* = 3). Different letters between bars indicate significant differences at *P* < 0.05 (Duncan’s range test).

Indole-3-acetic acid contents of ovaries significantly increased after spraying with an IAA solution, but parthenocarpy did not occur. The MT treatment did not significantly increase the IAA content, while hand pollination decreased the IAA content (**Figure 3A** and Supplementary Figure S5). Thus, IAA was not the key factor in pear fruit set for parthenocarpy. The ABA content of MT-treated ovaries was significantly lower than in the CK group at 5 DAA. MT, as well as hand pollination, could reduce ABA content, thereby promoting fruit set (**Figure 3B** and Supplementary Figure S5). On the 5th day, no GA_3_ was detected in the CK group, whereas the contents of bioactive GA_3_ in the ovaries increased significantly after the MT treatment (**Figure 3C** and Supplementary Figure S5). After the HP treatment, the GA_4_ content increased to 145 ng g^−1^ fresh weight (FW), 13-fold higher than in the CK, while GA_4_ content was twofold higher than in the CK after MT treatment (**Figure 3D** and Supplementary Figure S5). We thus speculated that MT induces the production of bioactive GAs.

**FIGURE 4:**
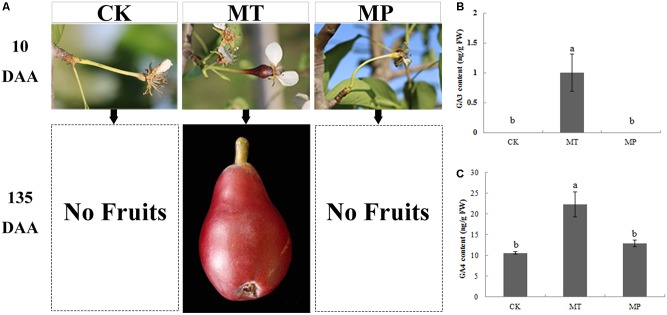
Effects of melatonin on fruit shape and gibberellin (GA_3_ and GA_4_) contents of pear ovaries after different treatments. CK, control; MT, melatonin treatment; MP, paclobutrazol plus melatonin treatment. Effects of melatonin on **(A)** fruit shape, **(B)** GA_3_ content, and **(C)** GA_4_ content.

We also studied the relationship between MT and GA using PAC. After treatment with PAC, spraying with MT did not lead to parthenocarpy (**Figure 4A**). The level of GA was significantly inhibited, GA_3_ was not detected in ovaries, and the GA_4_ contents reached 12.87 ng g^−1^ FW, which was consistent with the CK group. Thus, PAC inhibited GA synthesis, and GA is downstream of MT in the regulatory process of parthenocarpy (**Figures 4B,C**).

To obtain a general overview of the ‘Starkrimson’ transcriptome during MT- and MP-treatments and hand pollination, ovaries were harvested at 5 DAA. We obtained 41,845,974–59,518,366 clean reads from the samples, and more than 69% of the clean reads were mapped to the reference pear (*Pyrus bretschneideri* Rehd.) genome (Supplementary Table S2).

**FIGURE 5:**
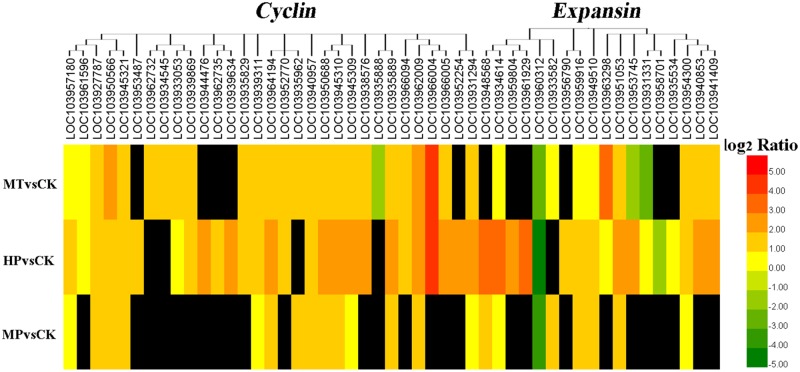
Expression profiles of cyclin and expansin family genes. The black box indicates no significant differences between treatments.

Differentially expressed genes (DEGs) were grouped based upon their biological functions using MapMan to analyze carbohydrate and photosynthesis-related metabolisms. Photosynthesis-related genes were activated, with 183 upregulated DEGs in pollinated ovaries and 144 upregulated DEGs in MT-induced parthenocarpic fruit. After blocking GA synthesis, however, only 60 upregulated DEGs were detected in ovaries (Supplementary Figure S6 and Supplementary Table S5). Similarly, cell division-related and cell expansion-related genes were upregulated in MT-induced parthenocarpic fruit, as well as in pollinated ovaries (Supplementary Table S6). With the exception of a downregulated *PbCyclinA1-1* gene, the differentially expressed cyclins were upregulated in MT-treated ovaries at 5 DAA. *CyclinA2* was upregulated more than fourfold (log2 fold change) in both MT-treated and pollinated ovaries. RNA sequencing showed that 12 and 17 expansion genes were regulated during MT and HP treatments, respectively, of which 9 and 15 genes, respectively, were upregulated (**Figure 5** and Supplementary Table S6). Six unigenes were chosen for quantitative PCR from cyclin- and expansion-related genes, and they exhibited similar expression tendencies as determined by the sequencing results (Supplementary Figure S7). Thus, MT can regulate carbohydrate and photosynthesis-related metabolisms and also promotes cell division and cell expansion in the early fruit setting stage.

**FIGURE 6:**
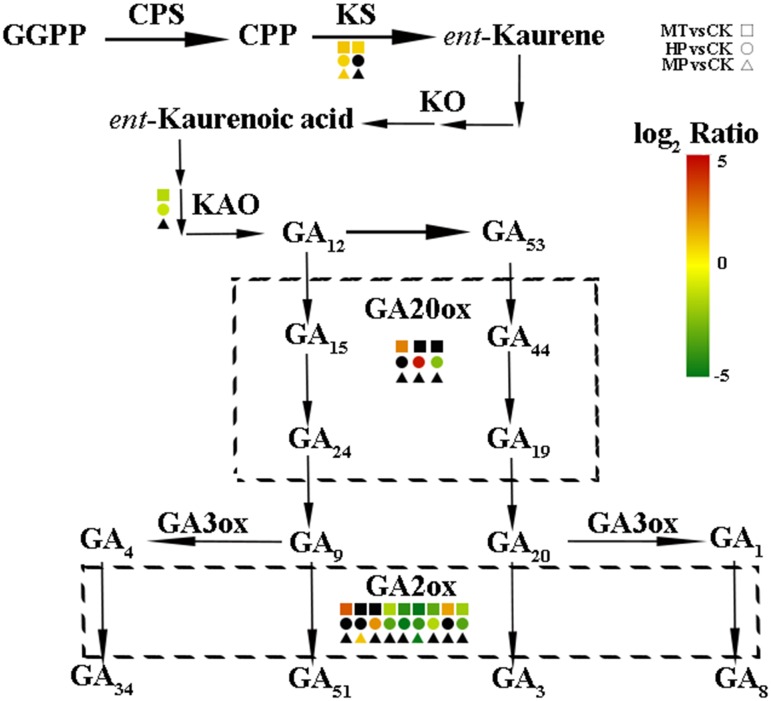
Expression levels of gibberellin biosynthesis-related genes in pear ovaries after different treatments 5 DAA. The black box indicates no significant differences between treatments.

**FIGURE 7:**
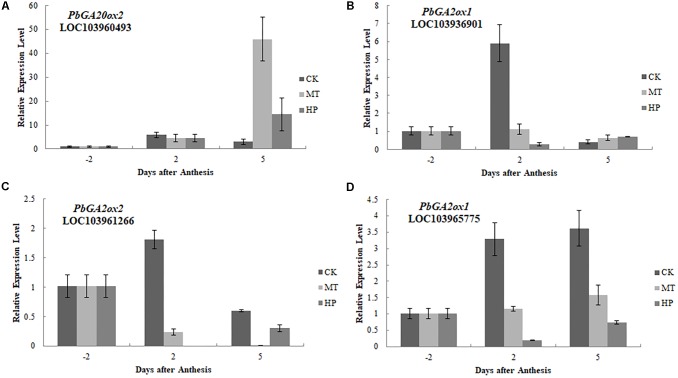
Expressions of gibberellin biosynthesis-related genes after different treatments. The expression level of each gene in ovaries under control conditions was normalized as 1.0. The results shown are means ± SE (*n* = 3).

To further investigate the regulatory mechanisms of MT, we analyzed changes in the expression of phytohormone-related genes in the transcriptome. A number of genes in the GA biosynthesis pathway were induced. Two genes encoding ent-kaur-16-ene synthase were significantly upregulated in MT- and HP-treated ovaries (**Figure 6**). MT led to the upregulation of *PbGA20ox2* (LOC103942611), to twofold higher than in the CK, while HP caused a significant upregulation of *PbGA20ox2* (LOC103960493), to fourfold higher than in the CK. In MT and HP treatments, five *GA2ox* genes showed significant downregulation, among which *PbGA2ox* (LOC103951277) was downregulated sevenfold in the HP treatment and fourfold in the MT treatment, while *PbGA2ox2* (LOC103956941) was downregulated fivefold in the MT treatment and fourfold in the HP treatment. In the MP group, only one *GA2ox* was downregulated, and no significant differences were observed between the other *GA2oxs* and those of the CK group (**Figure 6** and Supplementary Table S3). Four GA synthesis-related unigenes were chosen for quantitative PCR, and they exhibited similar expression tendencies as those determined by the sequencing results. No differences in *PbGA20ox2* expression was detected at 2 DAA, but *GA2oxs* showed differential expression levels at 5 DAA (**Figure 7**). To conclude, MT mainly downregulated the expression of the *GA2oxs* in the GA synthesis pathway, resulting in the synthesis of bioactive GA. Auxin- and ABA-mediated signaling pathways were also activated. In total, 106 and 96 DEGs involved in auxin and ABA signal transduction, respectively, were also modified (Supplementary Figure S8 and Supplementary Table S4).

## Discussion

Parthenocarpy, the production of seedless fruit without fertilization, can be either natural or artificial. Parthenocarpy is highly beneficial, as insects or pollinizers are not needed to produce seedless fruits that are popular with consumers. Plant hormones such as auxin can induce parthenocarpy in various plants, including cucumber, tomato, and muskmelon (Mariotti et al., 2011). According to our results, exogenous MT can also induce parthenocarpy in pear.

Melatonin acts as a growth promoter in many species, functioning in a similar manner as IAA (Chen et al., 2009; Park and Back, 2012). Interestingly, we found that exogenous MT induced parthenocarpy in pear, whereas exogenous IAA could not (**Figure 1**). Previous studies have shown that ovaries synthesize large amounts of IAA post-pollination to promote fruit development (Bermejo et al., 2015). In our study, IAA content as well as the number of seed cell layers in the ovary did not increase after MT treatment, and IAA content declined after pollination compared with the control. These results contradict other results in which IAA induced parthenocarpy in tomato and eggplant (Du et al., 2016). Auxin-mediated signaling pathway-related genes did not change consistently after MT treatment. These results indicate that IAA is not a major factor in pear parthenocarpy.

Photosynthesis and carbohydrate metabolism provide necessary nutrition for fruit and seed set. In tomato, expression levels of photosynthesis-associated genes are upregulated during the parthenogenetic process (Wang et al., 2009). Our analysis corroborated those results, as most photosynthesis- and carbohydrate-related genes were upregulated after MT treatment. In regards to photosystems I and II, chlorophyll-related genes were significantly upregulated, with chlorophyll a-b binding protein and photosystem I reaction center subunit also significantly upregulated under MT induction. In contrast, DEGs were significantly reduced after pretreatment with PAC. Among carbohydrate-related genes, starch synthase genes were downregulated, sucrose synthase genes were upregulated, and genes related to sucrose degradation enzymes were significantly downregulated. These patterns may be related to the accumulation of nutrients during fruit development. In the MP-treated group, however, the number of DEGs was significantly reduced. This result suggests that MT regulates the mechanisms of photosynthesis and carbohydrate induction through the GA pathway (Supplementary Table S5). As revealed by vascular bundle staining, nutrients of unpollinated plants could not be transported to the ovary at 10 DAA and thus could not promote normal fruit development. In MT and HP groups, however, plant vascular bundles remained intact and could therefore transport nutrients normally. In addition to proper nutrient uptake, MT can further promote cell division and cell expansion, a conclusion confirmed by cell histology observation. At the transcriptome level, plant cyclin genes are important in regulating the commitment of cells to cell division during plant growth and development (Riou-Khamlichi et al., 1999). In Arabidopsis, the overexpression of *AtCYCD* genes enhances cell division and accelerates plant development (Collins et al., 2012). Expansin A and B belong to the α- and β-expansin families, respectively, and many of their members have the ability to induce rapid cell expansion (Kende et al., 2004). In tomato and litchi, expansin genes are differentially expressed in growing and ripening fruit, suggesting that expansins are involved in fruit growth and development (Brummell et al., 1999). Thus, plant cyclins and expansins play important roles in cell division and plant development (Choi et al., 2006). Transcriptome data showed that the numbers of cell cycle- and cell expansion-related genes were modified by MT treatment. In particular, cyclins, except for *PbCyclinA1-1*, were upregulated, and 9 of 12 differentially regulated expansins were upregulated. After PAC treatment, the number of DEGs in MP-treated samples was significantly reduced, which suggests that MT can promote cell division and cell expansion through the GA pathway (**Figure 5** and Supplementary Table S6).

Gibberellins play important roles during parthenocarpy and fruit development in pear (Niu et al., 2015). In different plant species, the overexpression of GA-biosynthesis genes leads to the following characteristic phenotype: longer hypocotyls and roots, and taller plants with longer and thinner internodes (Eriksson et al., 2000; Vidal et al., 2001). In pear after MT treatment in our study, GA_3_ and GA_4_ contents of ovaries increased, the fruit shape index increased significantly, fruit transverse diameter decreased and the longitudinal diameter obviously increased, all effects similar to those of GA treatment (**Figure 3** and Supplementary Figure S4). C20-GAs have a full complement of 20 carbon atoms, whereas C19-GAs possess only 19 carbon atoms, having lost carbon-20 by metabolism. C20-GAs do not normally have any biological activity, but they can be metabolized to C19-GAs that may be bioactive. C19-GAs include GA_3_ and GA_4_ (Sponsel and Hedden, 2010). MT may thus increase bioactive GAs (GA_3_ and GA_4_) during the parthenocarpy process. Zhang et al. (2014) have also confirmed that MT can increase the GA_4_ content of cucumber under high salinity stress, a finding in agreement with our results. PAC has been used to study the interaction mechanisms of different hormones and GAs in tomato and Arabidopsis (Ding et al., 2015; Zhang et al., 2017). After treatment with PAC, IAA-induced parthenocarpy is greatly reduced in tomato (Serrani et al., 2008). In our study, MT could induce parthenocarpy; after PAC pretreatment, however, MT did not lead to parthenocarpy, which indicates that GA is downstream of the regulatory process. Many key enzymes genes in the GA synthesis pathway are differentially regulated during parthenocarpy. Expression levels of *GA20ox* and *GA3ox*, which promote GA synthesis, are increased after pollination but are significantly decreased in unpollinated ovaries (Talon et al., 1992; Fos et al., 2001). Moreover, overexpression of *GA20ox* in citrus and tomato can induce parthenocarpy (Garcíahurtado et al., 2012). *GA2ox* can inactivate GA_1_, GA_4_ and their precursors (Rieu et al., 2008), all of which are involved in the main GA-inactivating process. *GA2ox* plays important roles in regulating GA levels in ovaries and axillary buds, and silencing this gene can promote parthenocarpic fruit growth (Martínez-bello et al., 2015). *GA20ox* and *GA2ox* are the key genes with important functions in parthenocarpy. In our study, MT upregulated the expression of *GA20ox*, whereas the expression of *GA2ox* was downregulated in MT and HP groups. In the MP group, no significant changes in the key genes of the GA synthetic pathway were observed compared with the CK (Supplementary Table S3). We thus infer that MT significantly promotes GA biosynthesis by affecting *GA20ox* and *GA2ox* genes, thereby inducing parthenocarpy.

## Conclusion

**FIGURE 8:**
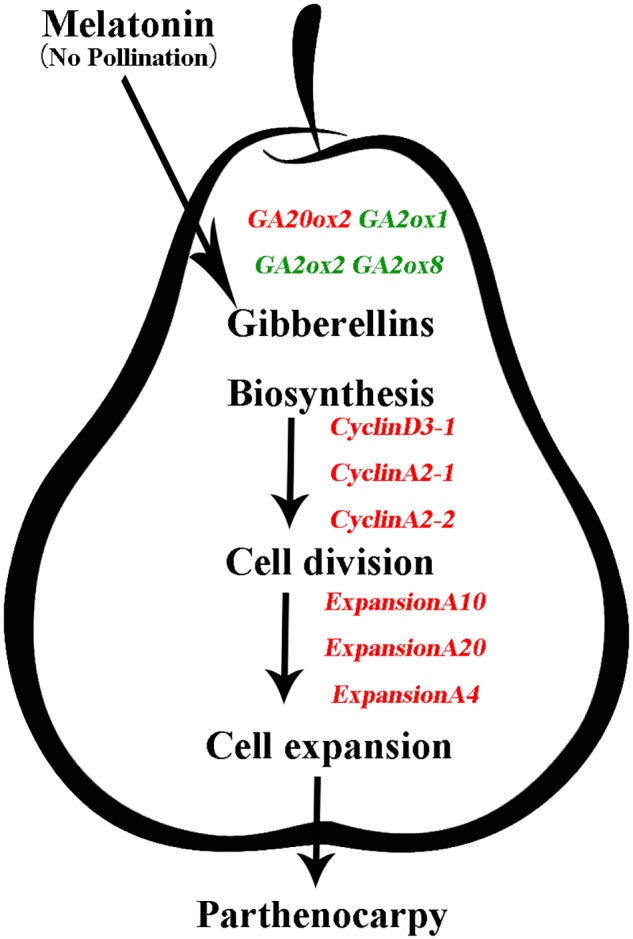
Model depicting the mechanism of gibberellins (GAs) in melatonin-mediated fruit set in ‘Starkrimson’ pear. Exogenous melatonin is involved in plant hormone metabolism, and melatonin regulates the process of parthenocarpy through the GA-synthesis pathway. *GA20ox* and *GA2ox* genes are mainly regulated to increase GA content, which promotes fruit set, and melatonin triggers cell division and expansion of the mesocarp to form fruit through induced GA biosynthesis.

In this study, we demonstrated that exogenous MT induces parthenocarpy in pear (**Figure 2**). Moreover, we found that exogenous MT is involved in plant hormone metabolism and that MT regulates the process of parthenocarpy via the GA synthesis pathway. *GA20ox* and *GA2ox* genes are mainly regulated to increase GA content and promote fruit set, with MT triggering cell division and expansion of the mesocarp to form fruit by inducing GA biosynthesis (**Figure 8**). To the best of our knowledge, the present study is the first attempt to elucidate the mechanisms by which MT induces parthenocarpy in pear.

## Author Contributions

JL, ZW, and RZ designed the experiments. JL, LL, HW, FL, and YZ performed the experiments. JL analyzed the data. JL, ZW, CY, FM, and LX wrote and revised the manuscript. All authors have participated in this research and approved the final manuscript.

## Conflict of Interest Statement

The authors declare that the research was conducted in the absence of any commercial or financial relationships that could be construed as a potential conflict of interest.

## Footnotes

1 http://www.ncbi.nlm.nih.gov/genome/?term=pyrus
