# Supplementary material for: Melatonin Induces Parthenocarpy by Regulating Genes in Gibberellin Pathways of ‘Starkrimson’ Pear (Pyrus communis L.)
Source: Front Plant Sci. 2018 Jul 4;9:946. doi: 10.3389/fpls.2018.00946 (PMC6040045; doi:10.3389/fpls.2018.00946)
Supplement: Supplementary file 7 [file Data_Sheet_1.DOCX]

Figure S1.


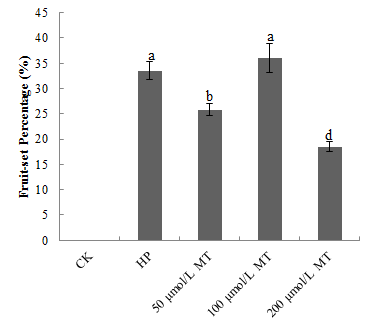


Supplementary Fig. S1 | The effects of melatonin on ‘Starkrimson’ fruit-set percentage.

Figure S2.


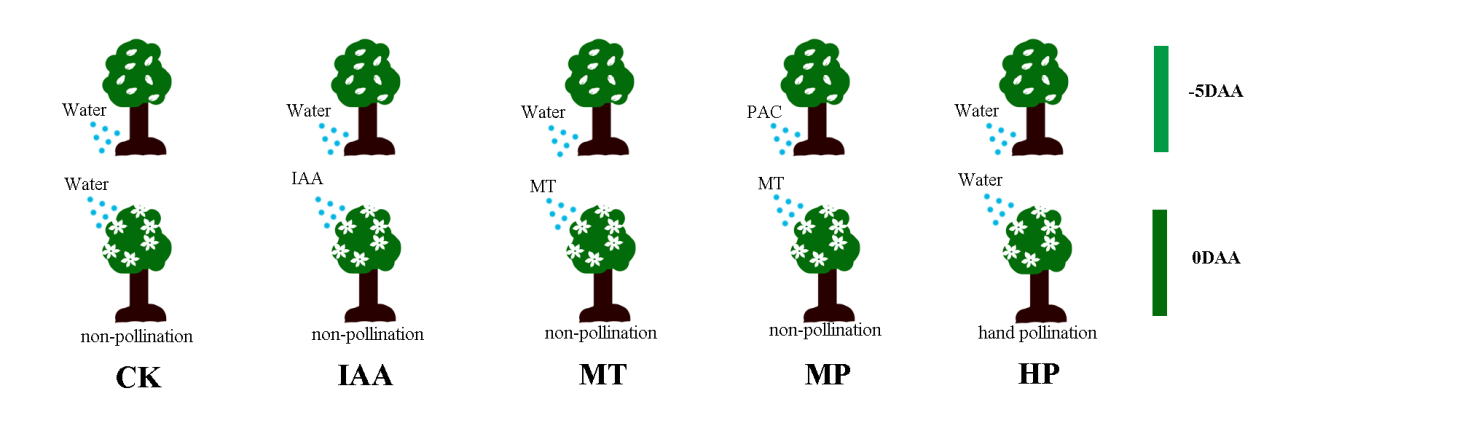


Supplementary Fig. S2 | Schematic representation of the experimental design. The whole inflorescence was bagged before and after flowering on April 1 to prevent natural pollination. Five days before the flowering period, pear trees were treated with water and PAC solution. Unpollinated inflorescences were treated with water, IAA, or by spraying with MT (CK, IAA and MT groups, respectively) at the flowering stage, and PAC-pretreated plants were treated by spraying with MT (MP) or subjected to artificial pollination (HP).

Figure S3.


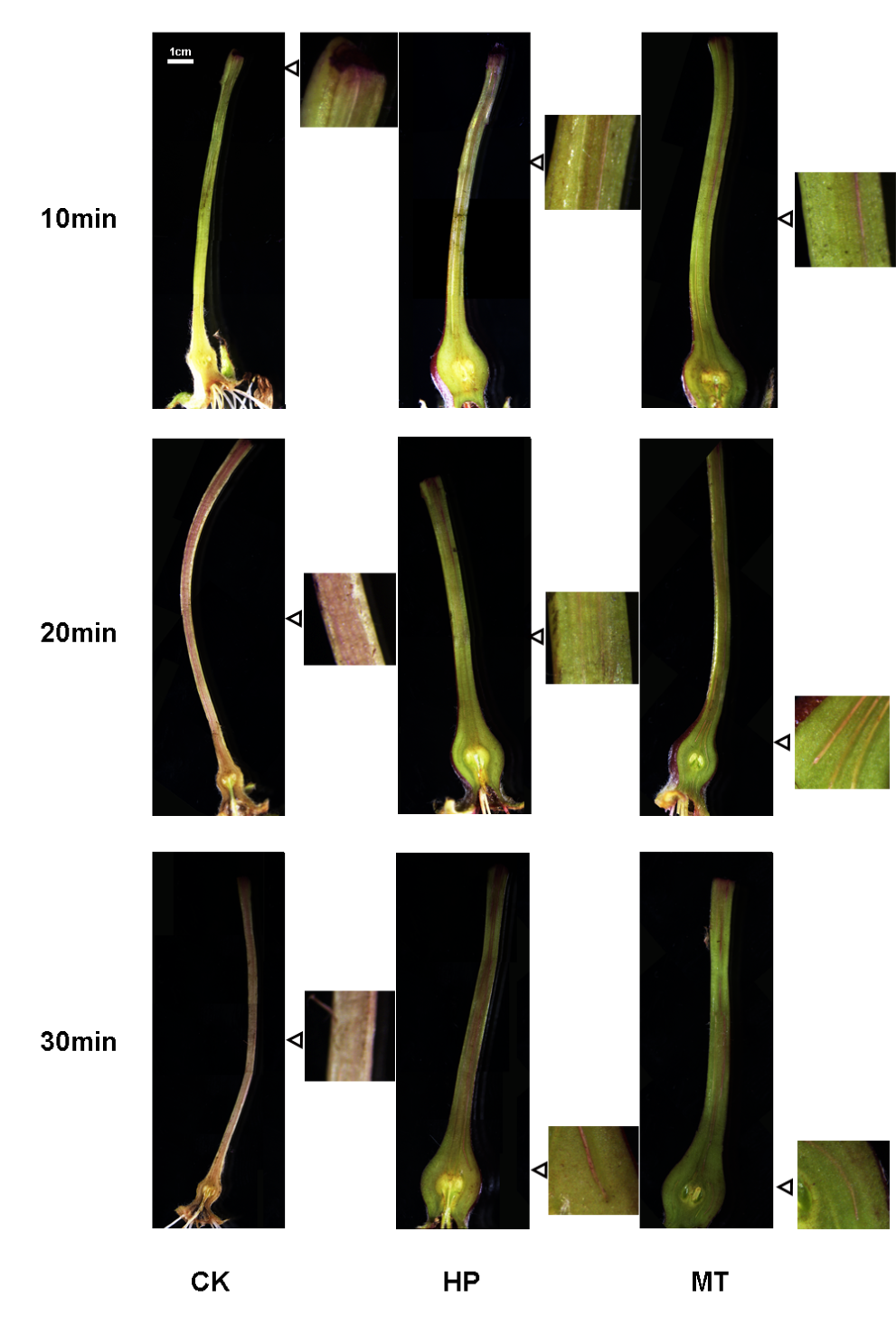


Supplementary Fig. S3 | Observations of vascular bundles of pear fruitlets stained with basic fuchsin 10 days after anthesis.

Fig. S4


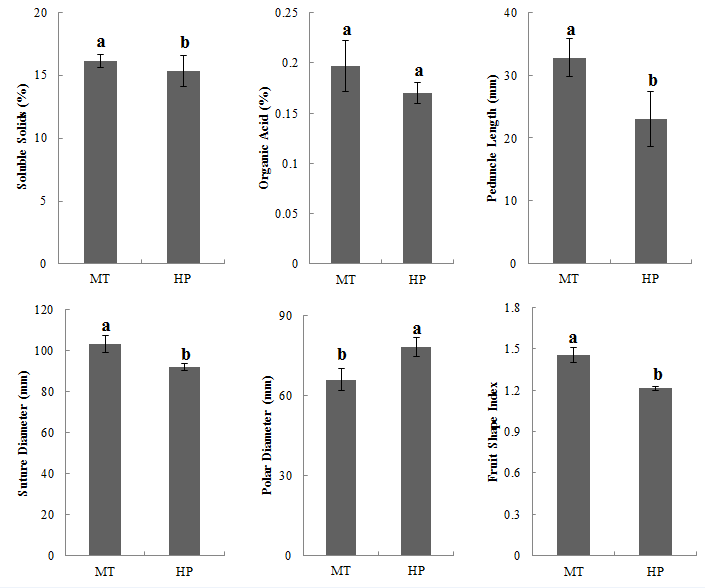


Supplementary Fig. S4 | Effects of exogenous melatonin on fruit quality and morphology 10 days after harvest. Results are means ± SD (*n* = 3). Different letters above bars indicate significant differences at *P* < 0.05 (Duncan’s range test).

Fig. S5


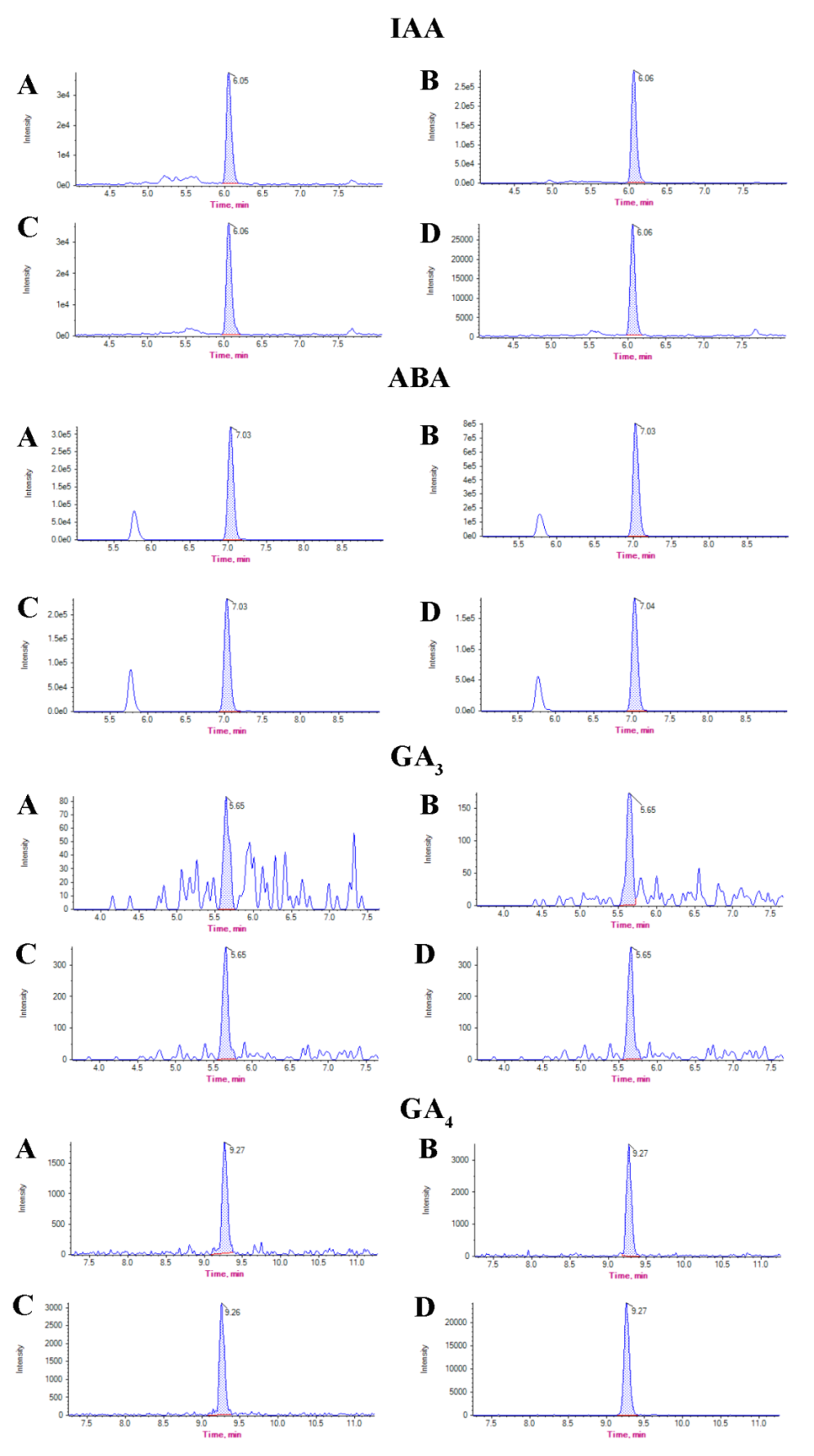


Supplementary Fig. S5 | HPLC chromatograms of indole-3-acetic acid (IAA), abscisic acid (ABA) and gibberellin (GA_3_ and GA_4_) determinations. A: control (CK); B: IAA treatment; C: melatonin treatment; D: hand pollinated.

Fig. S6


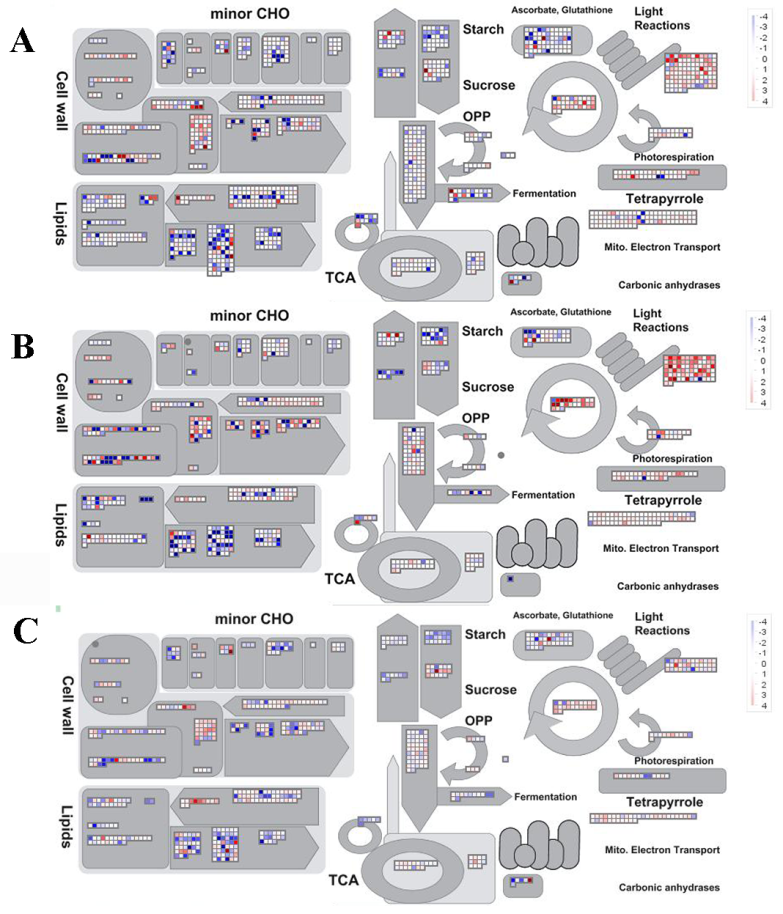


Supplementary Fig. S6 | Overview of photosynthesis and carbohydrate metabolism using a MapMan pathway. The ‘photosynthesis and carbohydrate metabolism overview’ MapMan pathway was used to visualize transcriptional changes in melatonin-induced ovaries (A), hand-pollinated ovaries (B) and melatonin-induced ovaries after paclobutrazol treatment (C) at 5 days after anthesis.

Fig. S7


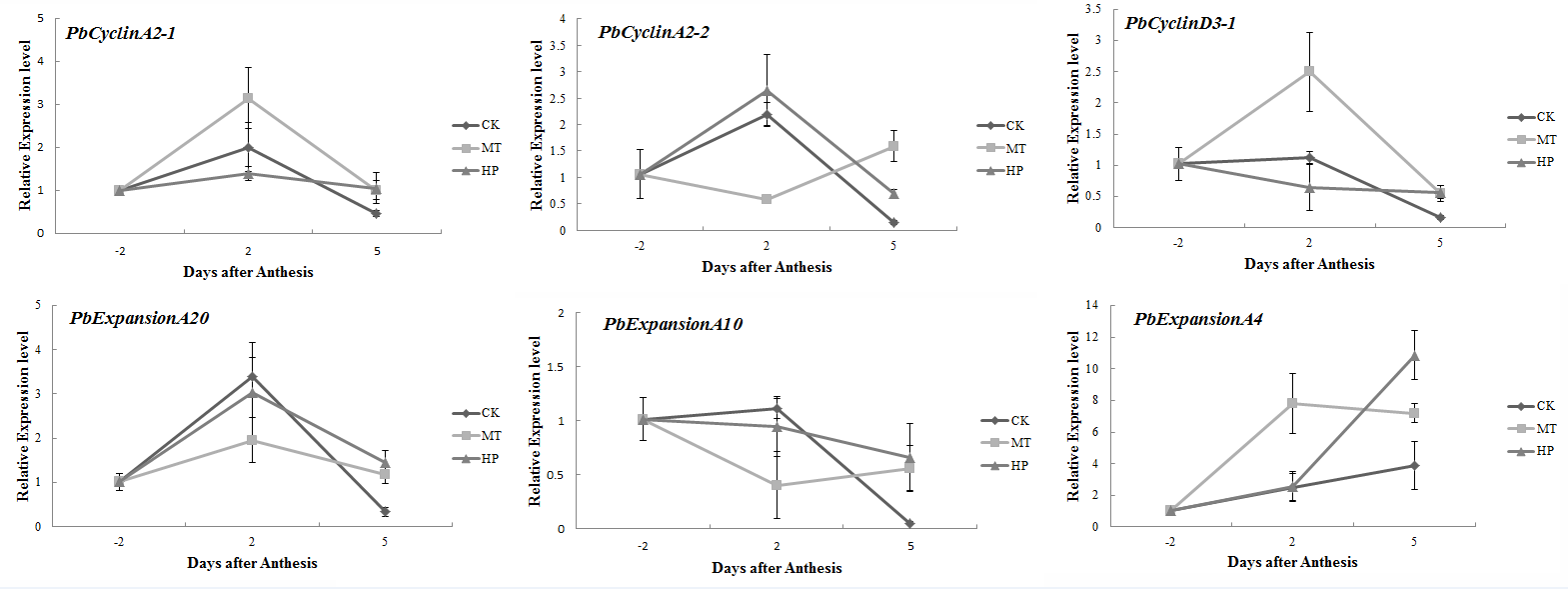


Supplementary Fig. S7 | Cell division- and cell expansion-related gene expressions after different treatments. The expression level of each gene in ovaries under control conditions was normalized as 1.0. The results shown are means ± SD (*n* = 3).

Fig. S8


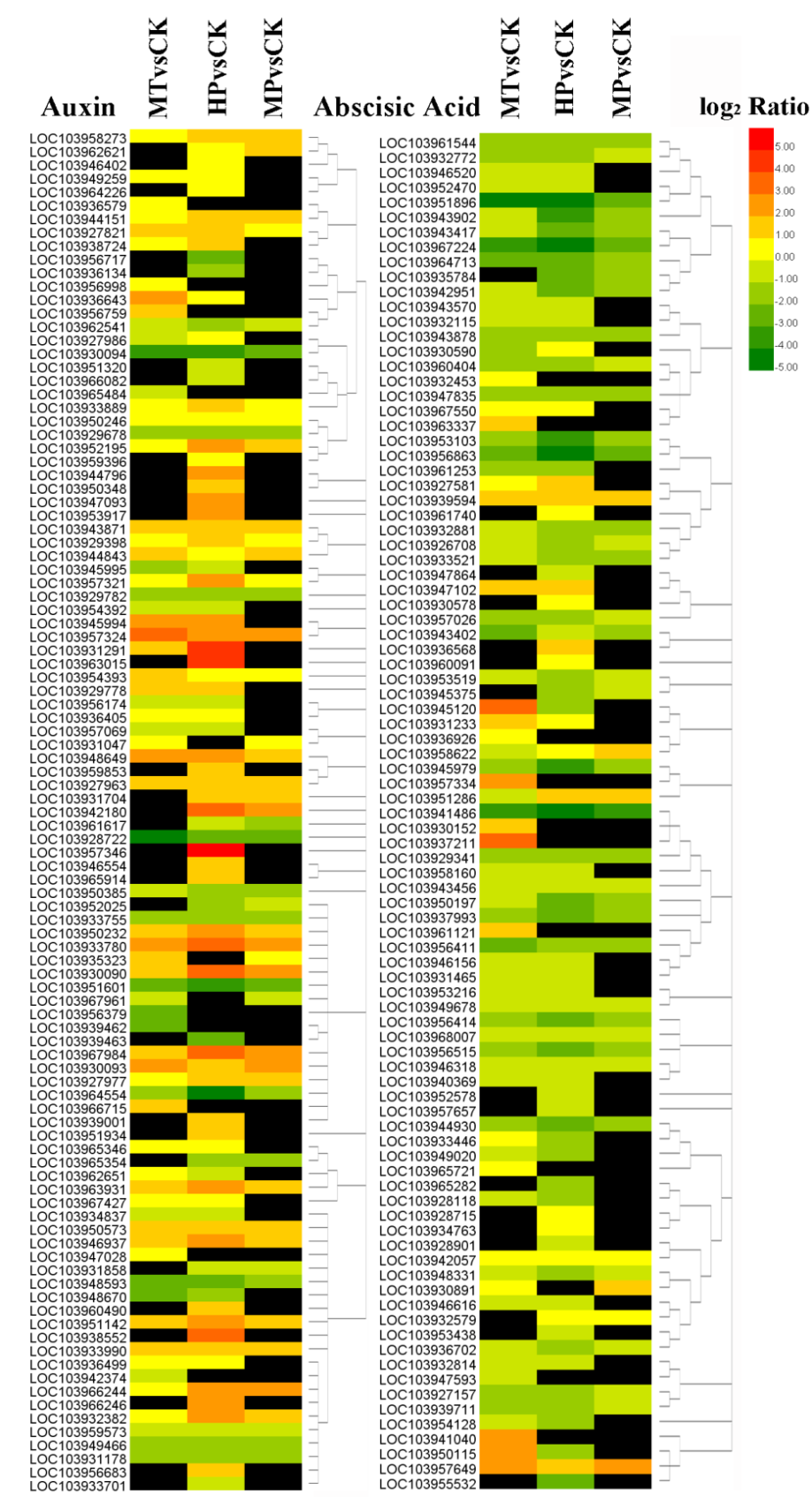


Supplementary Fig. S8 | Relative expressions of auxin- and abscisic acid-related genes in pear ovaries at 5 days after anthesis after different treatments. The black box indicates no significant differences between treatments.
